# *Pediococcus pentosaceus* MZF16 Probiotic Strain Prevents In Vitro Cytotoxic Effects of *Pseudomonas aeruginosa* H103 and Prolongs the Lifespan of *Caenorhabditis elegans*

**DOI:** 10.3390/pathogens14030244

**Published:** 2025-03-03

**Authors:** Meryem Boujnane, Mohamed Zommiti, Olivier Lesouhaitier, Mounir Ferchichi, Ali Tahrioui, Amine M. Boukerb, Nathalie Connil

**Affiliations:** 1 CBSA UR 4312, Université de Rouen Normandie, Université de Caen Normandie, Normandie Université, F-76000 Rouen, France; meryem.boujnane1@univ-rouen.fr (M.B.); mohamed.zommiti@hotmail.fr (M.Z.); olivier.lesouhaitier@univ-rouen.fr (O.L.); ali.tahrioui@univ-rouen.fr (A.T.); amine.boukerb@univ-rouen.fr (A.M.B.); 2 Unité de Protéomique Fonctionnelle et Potentiel Nutraceutique de la Biodiversité de Tunisie, Institut Supérieur des Sciences Biologiques Appliquées de Tunis, Université Tunis El-Manar, Tunis 1006, Tunisia; mounir.ferchichi@yahoo.fr

**Keywords:** *Pediococcus pentosaceus*, anti-virulence, *Pseudomonas aeruginosa*, quorum sensing, probiotic, *Caenorhabditis elegans*

## Abstract

*Pseudomonas aeruginosa* is an opportunistic pathogenic bacterium, responsible for several life-threatening infections due to its multiple virulence factors and problematic multi-drug resistance, hence the necessity to find alternatives such as competitive probiotics. *Pediococcus pentosaceus* MZF16 is an LAB strain, isolated from traditional dried meat “Ossban”, with high probiotic potential. Our study investigated the capacity of *P. pentosaceus* MZF16 to counteract *P. aeruginosa* H103 using several tests on intestinal cells (analysis of cytotoxicity, inflammation, adhesion/invasion) and on the in vivo *Caenorhabditis elegans* model. The effect of MZF16 on the *quorum sensing* of the pathogen was also examined. We found that *P. pentosaceus* MZF16 was able to reduce H103 cytotoxicity and inflammatory activity and prevented pathogen colonization and translocation across Caco-2/TC7 cells. MZF16 also exerted an anti-virulence effect by attenuating *quorum-sensing* (QS) molecules and pyoverdine production and extended *C. elegans* lifespan. The obtained results highlight the potential of *P. pentosaceus* MZF16 probiotic strain as an anti-*Pseudomonas aeruginosa* alternative and establish a basis for elucidating the mechanisms of *P. pentosaceus* MZF16 involved in countering *P. aeruginosa* virulence.

## 1. Introduction

Fermented foods are produced through controlled microbial growth and enzymatic conversion of food components. Historically, food fermentation was a common preservation method, used to maintain food integrity and reduce the risk of contamination with pathogenic microorganisms, likely due to the production of antimicrobial metabolites such as organic acids, ethanol and bacteriocins [1]. In recent years, fermented foods have surged in popularity, because of their health-promoting properties, especially for gastrointestinal health. These benefits are attributed to various mechanisms, including bioactive peptides and phenolic compound conversion to biologically active compounds, as well as the beneficial effects of constituent microorganisms [2]. Fermented foods such as dairy products, fermented meats and vegetables contain relatively resilient and stable microbial ecosystems, predominantly composed of lactic acid bacteria (LAB) and their primary metabolites including lactic acid [3]. Some LAB strains have long been recognized as probiotics due to their historical use and classification as beneficial bacteria with Generally Recognized As Safe (GRAS) status. Emerging probiotics are also being studied to better understand their potential health benefits. Among GRAS probiotics, the most well-known and studied bacteria belong to the Lactobacilli, recently reclassified into 23 genera [4] and various species including, for example, *Lacticaseibacillus casei*, *Lactobacillus acidophilus*, *Lactobacillus johnsonii*, and *Lactiplantibacillus plantarum*. These bacteria have demonstrated various health benefits, anti-carcinogenic, anti-diabetic, antioxidant, and anti-inflammatory potential, and also antimicrobial effects against food-borne pathogens [5].

Despite their frequent presence in many fermented products (i.e., meat, fish, dairy, beer, wine, fruits, vegetables, and beverages) [6], probiotics from the genus *Pediococcus* have been less studied. This genus consists of several species, including *P. acidilactici*, *P. pentosaceus*, *P. damnosus*, *P. parvulus*, *P. inopinatus*, *P. halophilus*, *P. dextrinicus*, and *P. urinaeequi* [7]. Among these, *P. acidilactici* and *P. pentosaceus* probiotic strains are notable for their high production of bacteriocins (pediocins) and bacteriocin-like substances (BLSs) [8]. Indeed, pediocins and other postbiotic metabolites (e.g., lactic acid, biosurfactants) from some *Pediococcus* species have exerted significant antibacterial and anti-virulence activities against several food-borne/food spoilage pathogens, such as *Listeria monocytogenes* and *Salmonella enterica* and also against the multi-drug-resistant (MDR) pathogen *Pseudomonas aeruginosa* [9,10,11,12,13,14].

*P. aeruginosa* is known to be a potential cause of the spoilage of various foods, notably those with high water content and nutrient-rich foods, such as milk, meats, and some fruits and vegetables [15,16]. It is also an opportunistic Gram-negative pathogen, widespread in the environment and responsible for healthcare-associated infections and food-borne diseases [17]. In some cases, this germ has been associated with diarrheal disease, systemic infections, enterocolitis and digestive cancers [18,19]. Its ability to cause severe acute and chronic life-threatening infections is attributed to its multiple virulence factors [20]. Moreover, the high versatility and adaptability of this pathogen are supported by its large genome (6 to 8 Mb), with over 8% dedicated to regulatory genes, enabling it to evade a broad spectrum of antimicrobial agents [21]. Thereby, alternative treatments, such as competitive probiotics, are urgently needed [22].

Some studies have explored the effects of postbiotic LAB metabolites, such as lactic acid, bacteriocins and BLSs produced by various *Pediococcus* species (*P. pentosaceus* and *P. acidilactici*, etc.) on *P. aeruginosa* [11,12,14]. However, interactions between these two species and also with the human host remain poorly characterized.

The *P. pentosaceus* MZF16 strain has been previously isolated in our laboratory from the artisanal Tunisian meat, “Dried Ossban”, and investigated for its probiotic potential [23,24]. The aim of the present study was to examine the interactions between *P. pentosaceus* MZF16 and the pathogen *P. aeruginosa* H103, with a particular focus on the MZF16 strain’s ability to modulate the virulence of the pathogen and its interactions with the host.

## 2. Materials and Methods

### 2.1. Bacterial Strains and Culture Conditions

The bacterial strains used in this study were the pathogen *P. aeruginosa* H103, a prototroph derivative of PAO1 strain [25], and the potential probiotic *P. pentosaceus* MZF16 [26]. They were, respectively, grown at 37 °C under rotary shaking (180 rpm) in Luria–Bertani (LB) medium and in static conditions in Man Rogosa Sharpe (MRS) medium.

### 2.2. P. aeruginosa H103-gfp and P. pentosaceus MZF16-mCherry Construction

*P. aeruginosa* H103 was grown in LB medium until reaching an OD_600 nm_ of 0.5, then centrifuged at 8000× *g* for 10 min at 4 °C. The pellet was washed and resuspended in 300 mM sucrose buffer at a concentration of 10^10^ CFU/mL. A 100 µL aliquot of electrocompetent *P. aeruginosa* was mixed with 50 ng of plasmid pHC60 (gfp, tetR) [27] and electroporated at 1.8 kV in a 0.2 cm cuvette. Immediately after electroporation, 1 mL of LB medium was added, and the mixture was incubated for 2 h at 37 °C with shaking at 180 rpm *P. aeruginosa* H103-gfp transformants were selected on LB agar supplemented with Tetracycline (100 µg/mL).

For *P. pentosaceus* MZF16-mCherry construction, electrotransformation was performed using plasmid pRCR12 (mCherry, CmR), provided by Pr Paloma Lopez (Madrid, Spain), and following the method of Pérez-Ramos et al. (2018) [28]. Briefly, the bacterial pellet was washed and resuspended in 1 mL of lysozyme (2000 U/ mL) and incubated for 20 min at 37 °C. Subsequently, 50 μL of electrocompetent cells was mixed with 5 μL of pRCR12 plasmid (0.5 μg) and electroporated at 2.5 kV in a 0.2 cm cuvette. After electroporation, 500 µL of MRS medium was immediately added, and the cells were incubated for 2 h at 37 °C. *P. pentosaceus* MZF16-mCherry transformants were selected on MRS-agar supplemented with Chloramphenicol (10 μg/mL).

All bacterial strains used in this study are listed below (Table 1).

### 2.3. Caco-2/TC7 Cell Culture and Infection

Human colon adenocarcinoma Caco-2/TC7 cells provided by Chantret et al. (1994) [29] were cultured in Dulbecco’s Modified Eagle Medium (DMEM, Lonza, Basel, Switzerland) supplemented with 20% heat-inactivated fetal bovine serum (FBS, Sigma Aldrich) and penicillin/streptomycin (100 µg/mL). The cells were cultivated and incubated at 37 °C in 5% CO_2_ atmosphere, and the medium was regularly changed. Cells were passed after reaching approximately 90% of confluence [30].

For adhesion, invasion, cytotoxicity assays and IL-8 quantification, cells were seeded at a concentration of 1.10^5^/mL, approximatively, in 24-well plates treated for tissue culture and grown to confluence. For bacterial translocation assay and transepithelial resistance (TER) measurements, cells were seeded on inserts (6.4 mm diameter, 3 μm pore size, Falcon) until full differentiation (19–21 days).

Bacterial suspensions of *P. aeruginosa* H103 and its mixture with *P. pentosaceus* MZF16 (1:100) were prepared in DMEM without FBS and antibiotics. These suspensions were added to confluent or differentiated Caco-2/TC7 cells and incubated at 37 °C in 5% CO_2_ for 3 h for adhesion and invasion assays, and overnight for the other experiments.

### 2.4. Cytotoxicity Assay and Interleukin-8 Quantification

Cytotoxicity and inflammation assays were performed on confluent Caco-2/TC7 cells grown in 24-well culture plates. Following overnight infection with bacterial suspensions, the supernatants were collected from Caco-2/TC7 monolayers [31]. Then, cytotoxicity was assessed by measuring lactate dehydrogenase (LDH), a cytoplasmic enzyme released upon cell death, using an enzymatic assay (Cyquant Kit, Invitrogen, Waltham, MA, USA). The amount of IL-8 cytokine in the supernatants was quantified using the IL-8 Human ELISA Kit (Invitrogen), a solid-phase sandwich Enzyme-Linked Immunosorbent (ELISA) Assay. The concentration of IL-8 (pg/mL) was defined using a correlation of a standard curve and the optical density OD_450 nm_.

### 2.5. In Vitro Adhesion and Invasion Assay

Bacterial adhesion and invasion into intestinal Caco-2/TC7 cells were assessed following Xu et al.’s method [32], with slight modifications. For the adhesion assay, Caco-2/TC7 cells were infected with suspensions of *P. aeruginosa* H103 and mixture with *P. pentosaceus* MZF16 (1:100 ratio) for 3 h. After incubation, cells were washed twice with phosphate-buffer saline (PBS) to remove non-associated bacteria and then lysed with 0.1% Triton X100. The lysates were serially diluted and plated on Cetrimide agar to quantify adherent pathogenic bacteria.

For the invasion assay, after 3 h of infection, adherent and non-internalized bacteria were eliminated by treating the cells with 300 μg/mL of gentamicin for 1 h. Following treatment, the cells were lysed, and internalized bacteria were quantified by plating.

For the visualization of bacterial adhesion, Caco-2/TC7 cells were seeded on glass-bottom 24-well plates (Sensoplate, Greiner) and infected with fluorescent strains *P. aeruginosa* H103-gfp and *P. pentosaceus* MZF16-mCherry for 3 h. Afterward, Caco-2/TC7 cells were stained with DAPI (4′,6-diamidino-2-phenylindole) and visualized using confocal laser scanning microscopy (CLSM). GFP, mCherry and DAPI were excited at 488 nm, 543 nm and 405 nm, respectively, and fluorescence emissions were detected between 484 and 549 nm, 539 and 599 nm, and 404 and 478 nm.

### 2.6. Bacterial Translocation and Transepithelial Electrical Resistance (TER) Measurement

After an overnight infection of Caco-2/TC7 differentiated cells cultured on inserts, aliquots from the basolateral compartment were collected [33]. The number of translocated *P. aeruginosa* that crossed the epithelial monolayers was determined by serial dilution and plating on Cetrimide agar.

To assess the integrity of the epithelial barrier, the transepithelial electrical resistance (TER) of infected and non-infected monolayers was measured using the Millicell Electrical Resistance System (Millipore Corp, Bedford, MA, USA).

### 2.7. Antimicrobial Activity Test

The antagonistic activity of *P. pentosaceus* MZF16 against *P. aeruginosa* H103 was assessed using the disk diffusion method. Overnight cultures of both strains were centrifuged at 7000 *g* for 5 min. Supernatants of *P. pentosaceus* MZF16 were either pH-adjusted (pH = 7) or not before being filtered (0.22 µm membrane).

A suspension of *P. aeruginosa* H103 at a concentration of 10^6^ CFU/mL was spread on Muller Hinton Agar plate. Disks impregnated with cell-free supernatants (CFSs) of MZF16 (10 µL) were placed on the agar surface, and the plates were incubated at 37 °C for 24 h.

### 2.8. Bacterial Aggregation Assay

Auto- and coaggregation assays were performed as described previously [34] with minor modifications. Briefly, overnight cultures of H103 and MZF16 were centrifuged at 10,000× *g* for 10 min. The pellets were washed twice and resuspended in sterile phosphate-buffer saline (PBS). The bacterial suspensions were adjusted to an OD_600 nm_ of approximately 0.3.

For the coaggregation assay, equal volumes of the standardized bacterial suspensions were mixed. Both individual and mixed suspensions were aliquoted into hemolysis tubes (4 mL) and incubated at 37 °C without agitation.

The OD_600 nm_ of the suspensions was measured at the start of the experiment (A_0_) and after 3 h of incubation at 37 °C (A_t=3h_). The autoaggregation percentage was calculated as follows:

$$Autoaggregation\;\left(\%\right)=\left[1-\left(\frac{A_{t=3h}}{A_{0}}\right)\right]\;\times \;100$$

The coaggregation percentage was determined using the following formula:

$$Coaggregation\;\left(\%\right)=\left[\left(\frac{\left(A_{H103+MZF16}\right)-(A_{mix})}{\left(A_{H103+MZF16}\right)}\right)\right]\;\times 100$$

where A_H103_ and A_MZF16_ are the OD_600 nm_ values of the mixed bacterial suspension at time 0, and A_mix_ is the OD_600 nm_ of the mixed suspension at t = 3 h.

### 2.9. Pyoverdine Production Measurement

Pyoverdine is a key siderophore and virulence factor of *P. aeruginosa*. Its production was quantified by spectrophotometry at A_405 nm_ [35]. Bacterial suspensions of *P. aeruginosa* H103 and the mix with the probiotic *P. pentosaceus* MZF16 were grown in colorless DMEM medium supplemented with 20% fetal bovine serum (FBS). After incubation, pyoverdine production was measured from collected supernatants. Results are expressed as the relative pyoverdine levels (mean ± SEM) compared to the control, *P. aeruginosa* H103 culture.

### 2.10. Extraction and Quantification of AHL and HAQ Molecules

*N*-acyl-homoserine lactones (AHLs) and 2-alkyl-4-quinolones (HAQs, PQS system) signaling molecules were extracted from *P. aeruginosa* H103 cultures and cocultures with *P. pentosaceus* MZF16 (1:100), grown in DMEM medium, following the protocol described by Fletcher et al. (2007) [36]. For quantification, *Escherichia coli*-carrying plasmid pSB401 (*luxRI′::luxCDABE*) and *P. aeruginosa* PAO1 *ΔpqsA* CTX-*lux::pqsA* strain were used as biosensors for AHLs and HAQs, respectively. The assay was performed using a combined spectrophotometer/luminometer approach. Briefly, overnight cultures of the biosensor strains were adjusted to an OD_580 nm_ = 1 and then diluted to 1:50 and 1:100 ratios. Crude extracts of *quorum-sensing* (QS) molecules were diluted in LB medium and added to a 1:50 dilution of the biosensors. The 3-oxo-C12-HSL, C4-HSL, HHQ, and PQS synthetic standards (Sigma-Aldrich, Saint-Louis, MO, USA) at final concentrations of 5 μM were added to a 1:100 dilution of the biosensor strains as positive controls.

The assay was performed in 96-well microtiter plates (white-sided, clear-bottom). Bioluminescence (relative light units, RLU) and absorbance (A_580 nm_) were measured every 15 min for 24 h at 37 °C using a Spark 20 M multimode microplate reader (Tecan, Männedorf, Switzerland). The bioluminescence data were normalized to the A_580 nm_ values.

### 2.11. In Vivo Caenorhabditis elegans Killing Assay

The *C. elegans* slow killing assay was performed as previously described by Tan et al. [37]. *C. elegans* wild-type Bristol strain N2 worms were grown at 22 °C on nematode growth medium (NGM) agar plates using *Escherichia coli* OP50 strain as the nutrient. *P. aeruginosa* culture and its coculture with *P. pentosaceus* MZF16 were spread onto 35 mm NGM solidified agar plates before an overnight incubation at 37°. The plates were cooled to room temperature for 4 h. Then, 20 to 30 L4-synchronized worms prepared as described by Blier et al. [38] were plated and incubated at 22 °C in a humid environment to prevent plate drying. Worm survival was scored daily for 16 days using an optical microscope (Leica CME, Glattbrugg, Switzerland). The worms were considered dead when they remained static without grinder movements for 20 s or did not respond to light flashes. Results are expressed as a percentage of living worms. The results are the average of at least three independent assays. The nematode survival was calculated using the Kaplan–Meier method, and survival differences were tested for significance using the log–rank test (GraphPad Prism version 4.0, Dotmatics, San Diego, CA, USA).

### 2.12. Statistical Analysis

Statistical analysis of the data was performed using GraphPad Prism software version 10.2.2 with two-sample unpaired tests. All tests were conducted at least three times, and *t*-tests were used for all data analysis; statistical significance was considered at *p* < 0.05, *p* < 0.01 and *p* < 0.001.

## 3. Results

### 3.1. Cytotoxicity Assay and Interleukin-8 Quantification

**Figure 1:**
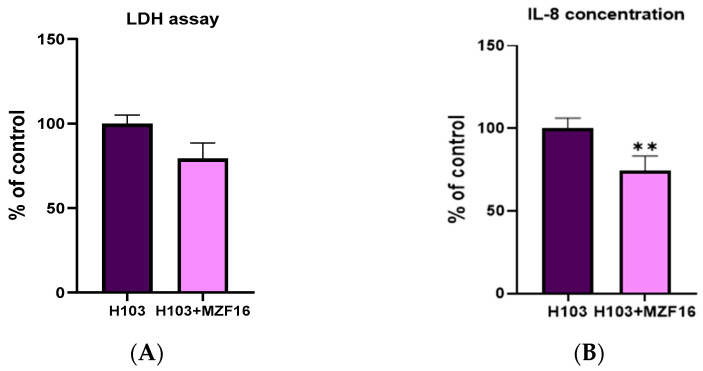
Effect of *P. pentosaceus* MZF16 on the cytotoxic activity of *P. aeruginosa* H103 toward Caco-2/TC7 cells (**A**) and on its pro-inflammatory potential and interleukin-8 (IL-8) secretion (**B**). Cytotoxicity was evaluated by measurement of lactate dehydrogenase (LDH) released from damaged cells after an overnight infection (** *p* < 0.01).

The cytotoxicity and pro-inflammatory activity of *P. aeruginosa* H103 towards the intestinal Caco-2/TC7 cells were evaluated after overnight incubation in the presence or absence of *P. pentosaceus* MZF16. The results demonstrated that MZF16 slightly decreased the level of lactate dehydrogenase (LDH) produced by the eukaryotic cells in response to the cytotoxic effect of the pathogen, with a reduction of about 20% (Figure 1A). In addition, MZF16 significantly reduced the level of IL-8 by more than 25% (Figure 1B).

### 3.2. Adhesion and Invasion

**Figure 2:**
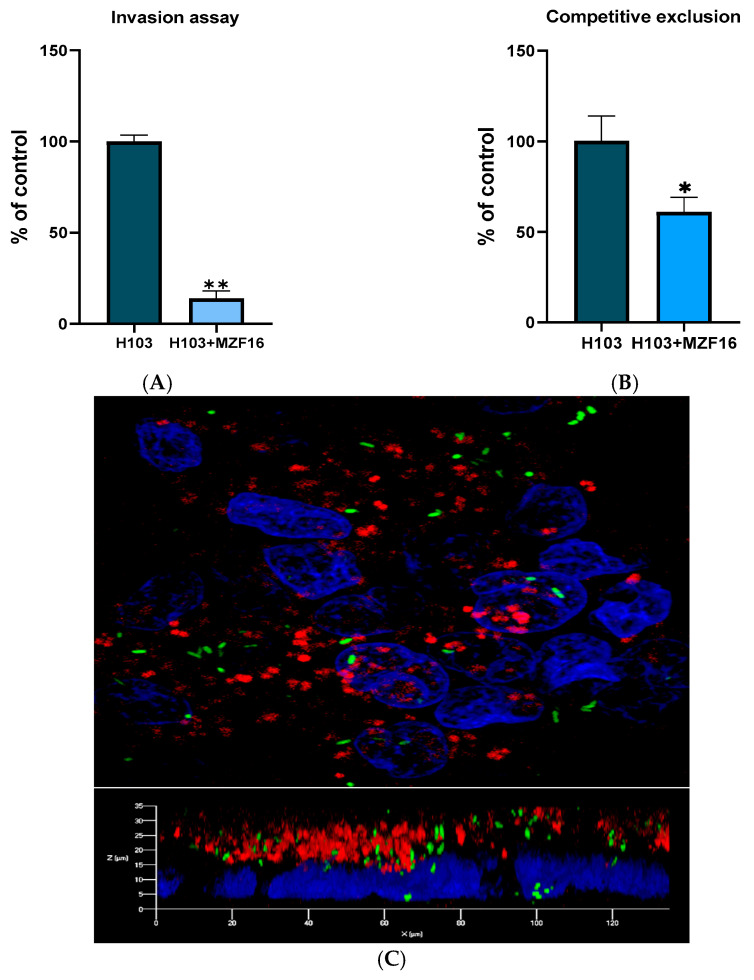
Inhibition of *P. aeruginosa* adhesion (**A**) and invasion (**B**) to Caco-2/TC7 cells by *P. pentosaceus* MZF16. The adhesion of the pathogen into Caco-2/TC7 cells in presence of *P. pentosaceus* MZF16 (1:100) was examined by plating cell lysates, after 3 h of contact, on Cetrimide agar, while invasive bacteria was selected by gentamicin (300 µg/mL) treatment for 1 h before plating the lysate. Each experiment was assayed at least four times (* *p* < 0.05, ** *p* < 0.01). (**C**) Three-dimensional CLSM visualization of H103-gfp and MZF16-mCherry adhesion to DAPI-colored Caco-2/TC7 cells. Observation was made using a 40× oil immersion objective.

The competitive exclusion of pathogen adhesion and invasion to Caco-2 cells by *P. pentosaceus* MZF16 was evaluated over 3 h by spreading cell lysates on Cetrimide agar. The simultaneous addition of *P. pentosaceus* MZF16 with *P. aeruginosa* H103 to intestinal Caco-2/TC7 cells resulted in a decrease of 39 ± 8% in the number of adhering *P. aeruginosa* H103 (Figure 2A), and a reduction of 85 ± 4% in invasive bacteria number (Figure 2B). Visualization of bacterial adhesion to Caco-2 cells using confocal laser scanning microscopy (CLSM) showed that MZF16-mCherry formed a layer on the cells. In contrast, H103-gfp appeared more scattered in the different stacks, likely due to its mobility and invasive ability (Figure 2C).

### 3.3. Transepithelial Electric Resistance (TER) and Translocation

**Figure 3:**
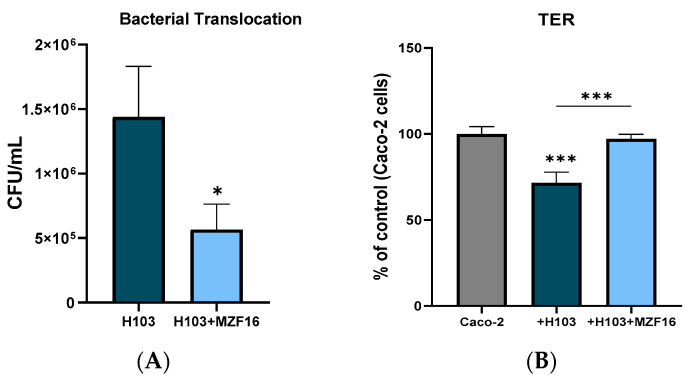
*P. pentosaceus* MZF16 effect on bacterial translocation of *P. aeruginosa* (**A**) and on Caco-2/TC7 differentiated cells transepithelial resistance (**B**). *P. aeruginosa* translocation was assessed by plating the bottom of the well on Cetrimide medium after an overnight infection of Caco-2/TC7 with the pathogen H103 strain and its coculture with *P. pentosaceus* MZF16 (1:100) (* *p* < 0.05, *** *p* < 0.001).

The transepithelial resistance (TER) and the bacterial translocation of *P. aeruginosa* H103 were evaluated on differentiated Caco-2/TC7 cells after an overnight infection. Results showed that *P. pentosaceus* MZF16 prevented the reduction in TER provoked by *P. aeruginosa* H103, which was about 30% (Figure 3A). Additionally, *P. pentosaceus* MZF16 significantly reduced the translocation of the pathogen across the Caco-2/TC7 cells by three times (Figure 3B).

### 3.4. Autoaggregation and Coaggregation

The autoaggregation and coaggregation abilities of *P. pentosaceus* MZF16 and *P. aeruginosa* H103 were measured in PBS after 3 h incubation at 37 °C. Both strains H103 and MZF16 demonstrated a high capacity of autoaggregation of about 43.8 ± 7.8 and 44.7% ± 2.6, respectively. Similarly, their coaggregation ability was about 45.7% ± 3.6.

### 3.5. Antimicrobial Activity Test

The cell-free supernatant of *P. pentosaceus* MZF16 exhibited no antibacterial activity against *P. aeruginosa* H103 in our conditions, suggesting the absence of an anti-*Pseudomonas* bacteriocin- or bacteriocin-like metabolite in our strain.

### 3.6. Quorum Sensing and Pyoverdine Production

**Figure 4:**
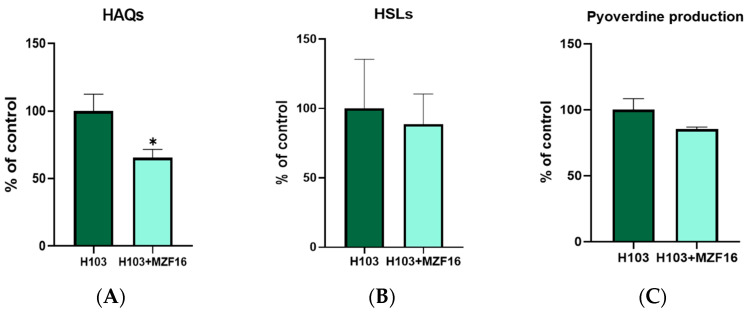
QS signaling molecules and virulence factors production. PQS molecules’ (HAQs) (**A**) and HSLs’ (**B**) production were quantified, in presence of *P. pentosaceus* MZF16 cells, comparing to the basic *P. aeruginosa* H103 production in DMEM medium. Bioluminescence measurements (± the SEM) normalized with A_600 nm_ along the bacterial growth of the AHL bioreporter strain, *E. coli* pSB401, and of the HAQ bioreporter strain, *P. aeruginosa* PAO1 *ΔpqsA pqsA::lux*. Histograms are a representation of both conditions at the peak of bioluminescence. The relative quantifications (± the SEM) of pyoverdine production (**C**) were determined by supernatants’ absorbance measurements at 405 nm. Each experiment was assayed at least four times independently. Statistics were determined by *t*-tests (* *p* < 0.05).

*N*-acyl-homoserine lactones (AHLs) production was assessed using the biosensor strain *E. coli* harboring the plasmid pSB401 (*luxRI′::luxCDABE*), able to detect short (C4) and long (C12) HSL chains specifically produced by *P. aeruginosa*. The production of 2-alkyl-4-quinolones (HAQs) signaling molecules (HHQ and PQS) was assessed using the *P. aeruginosa* PAO1 *ΔpqsA* CTX-*lux::pqsA* biosensor strain, which detects HAQ derivatives produced by *P. aeruginosa*. Both molecules’ production was examined in the presence and absence of the MZF16 strain. Results showed that *P. pentosaceus* MZF16 significantly reduced (of about 35%) the production of HAQ molecules by *P. aeruginosa* H103 (Figure 4A), but had no effect on HSL (Figure 4B) and pyoverdine production (Figure 4C).

### 3.7. In Vivo Virulence Test on C. elegans

**Figure 5:**
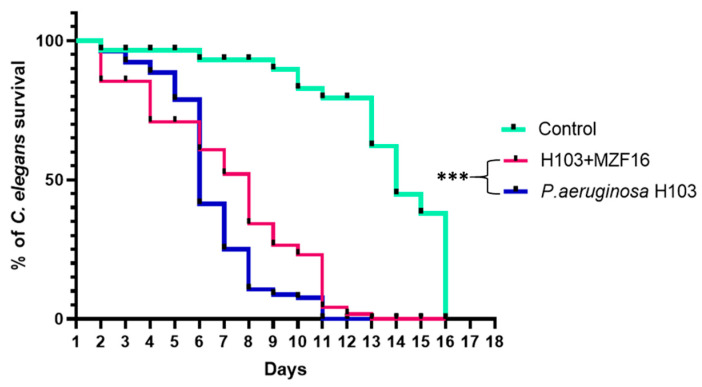
*C. elegans* nematode lifespan in contact with *P. aeruginosa* H103 (blue curve), and cocultures with *P. pentosaceus* MZF16 (inoculated in 1:100) (pink curve) ratio (*** *p* < 0.001). Untreated nematodes were used as control of basic survival (green curve). Experiment was conducted at least three times independently.

A slow killing assay was monitored over 16 days (Figure 5). The results showed that in the presence of *P. pentosaceus* MZF16, the lifespan of *C. elegans* was significantly prolonged compared to when the worms were exposed only to the pathogen *P. aeruginosa* H103. Specifically, on day 8 of the test, only 10% of the worms infected by H103 survived, while in the presence of *P. pentosaceus* MZF16, the survival rate of *C. elegans* infected by H103 was about 40%.

## 4. Discussion

*P. aeruginosa* is a major opportunistic pathogen responsible for both acute and chronic infections, especially in immunocompromised individuals [39]. The rise in multi-drug-resistant *P. aeruginosa* strains has led to an urgent need for alternative treatments to antibiotics, such as probiotics.

*P. pentosaceus* MZF16 strain, isolated from Tunisian dried meat (Ossban), exhibits probiotic properties, including resistance to the harsh gastrointestinal conditions and the high ability of adhesion to intestinal cells, without any risks for human health [23]. These traits underscore its potential in fighting pathogens.

In the present work, we evaluated the capacity of *P. pentosaceus* MZF16 to compete with *P. aeruginosa* H103 and reduce its virulence. For this, undifferentiated intestinal Caco-2/TC7 cells were treated simultaneously with both bacteria and the cytotoxicity, inflammatory potential, and adhesion/invasion of H103 have been investigated. Our study demonstrated that *P. pentosaceus* MZF16 can mitigate *P. aeruginosa* H103-induced cytotoxicity and inflammation in intestinal Caco-2/TC7 cells. Specifically, MZF16 reduced both LDH release (necrosis marker) and IL-8 secretion. These findings align with previous research showing similar protective effects of probiotics against various pathogens. For example, *P. acidilactici* SPM138 increased the human intestinal epithelial cells HT-29 cells’ survival rate from 20% to 98% and reduced by more than 60% IL-8 levels released upon *Clostridium difficile* infection [40]. Similarly, *P. acidilactici* TMAB26 demonstrated immunomodulatory effects by alleviating *Klebsiella pneumoniae* endotoxin-induced gut inflammation [12].

*P. pentosaceus* MZF16 also reduced the adhesion, invasion and translocation of *P. aeruginosa* H103 across undifferentiated/differentiated Caco-2/TC7 cells, preserving epithelial barrier integrity as evidenced by TEER measurements. Similar anti-adherence effects have been reported for *P. acidilactici* IAH-5 against *S.* Typhimurium [41]. In another study, *P. pentosaceus* L1 isolated from pickled radish also enabled the decrease in adhesion of an *E. coli* enterotoxigenic strain to porcine intestinal epithelial cells [42]. Likewise, *P. pentosaceus* 2–5 strain, isolated from chicken intestine, showed a strong ability to inhibit the adhesion of two enteropathogenic bacteria *E. coli* ATCC 25922 and *S.* Typhimurium ATCC 13311 to the epithelial Caco-2 cells, with the three anti-adhesion tested methods (competition, exclusion, substitution) [43].

The observed reduction in adhesion/invasion and cytotoxicity may result from direct probiotic–pathogen interaction or a postbiotic effect mediated by secreted factors. In fact, autoaggregation is stated to be a prerequisite for probiotic bacteria adhesion to intestinal epithelium, whereas their coaggregation abilities with pathogens enable forming an effective barrier, preventing epithelium colonization by harmful bacteria. Our strain *P. pentosaceus* MZF16 exhibited high autoaggregation (44.7%) and coaggregation abilities with H103 (45.7%), supporting its role in forming a protective barrier. This coaggregation capacity aligns with previous findings for *P. pentosaceus* DHR005 which showed lower autoaggregation (28.6%) and coaggregation (22%) against *Salmonella* Typhimurium PTCC1609 [41]. In a recent study, moderate to strong coaggregation abilities of two strains of *P. pentosaceus* H11 and H13, isolated from fermented bean curd, have been demonstrated with *P. aeruginosa* PAO1 [44], supporting our finding.

Since *P. pentosaceus* MZF16 is able to produce lactic acid and coagulin bacteriocin [23], we investigated if the protective effect of the probiotic was related to one of these aspects. Interestingly, despite the ability of *P. pentosaceus* MZF16 to produce antibacterial metabolites, no direct antimicrobial activity was detected against *P. aeruginosa* H103 under our experimental conditions. In contrast, Li et al. (2023) demonstrated that two strains *P. pentosaceus* H11 and H13 exhibited a different antibacterial effect on *P. aeruginosa,* suggesting that this antimicrobial ability was strain-specific [44]. In another studies, bacteriocins from other *Pediococcus* species; *P. acidilactici* and *P. inopinatus* strains exerted important antimicrobial activities against *P. aeruginosa* [10,14].

This lack of activity may be due to culture conditions, as bacteriocin production can vary based on time of exposition and media composition, notably the pH. Indeed, a comparative study has shown that no antibacterial activity against *P. aeruginosa* has been detected from pH-adjusted CFS of *P. pentosaceus* ENM104 comparing to the crude CFS, indicating that the production of antibacterial organic acids or bacteriocins may be active only under acidic conditions [45].

Nonetheless, we have demonstrated by spreading DMEM cocultured strains, that MZF16 does not inhibit H103 growth (Table S1). The ability of MZF16 to coexist with H103 suggests that in our experimental conditions, the protective effect of MZF16 was not directly linked to the production of lactic acid and/or the bacteriocin, but it may be related to a competitive mechanism for nutrients or epithelial binding sites [46].

Furthermore, other studies have shown that some *Pediococcus* species are able to produce exopolysaccharides and biosurfactants with a high ability to disrupt *P. aeruginosa* biofilms and display antimicrobial, anti-adhesive and anti-virulence properties [11,28].

The pathogenicity of *P. aeruginosa* involves the production of several virulence factors [47] such as pyoverdine and pyocyanin, regulated by the cell density-dependent signaling mechanism «*quorum sensing* (QS)» [48]. In *P. aeruginosa*, two main QS systems are defined, the system mediated by *N*-acylhomoserine lactones (AHLs) known as Las and Rhl, which are responsible for the production of homoserine lactone signal molecules, and the Pqs system depending on 2-alkyl-4-quinolones (HAQ) and which is interlinked with the Las and Rhl systems. These systems are involved in the pathogenesis of *P. aeruginosa* and the synthesis of some virulence factors such as rhamnolipid, pyocyanin, pyoverdine and the production of enzymes like proteases and lipases and biofilm formation [49,50].

The inhibition of this signalization can attenuate QS-related bacterial virulence [51,52]. Thus, anti-QS may be a promising strategy to fight against some pathogens including *P. aeruginosa*. In this context, we aimed to evaluate the effect of MZF16 on *P. aeruginosa* H103 QS. Interestingly, we found that the production of HSL molecules and PQS level were notably reduced in the presence of MZF16. Pyoverdine is a siderophore and an important virulence factor in *P. aeruginosa*, enabling iron sequestration from the host to improve the bacterial establishment during the infection. Our results showed that *P. pentosaceus* MZF16 decreased the pyoverdine level released in the coculture media, suggesting that *P. pentosaceus* MZF16 may be able to modulate the QS of *P. aeruginosa* H103. In agreement with our findings, *P. acidilactici* M7 have shown a *quorum quenching* activity, inhibiting short-chain HSL production, motility, and virulence factor production (elastase, protease, pyocyanin, and biofilm production) of *P. aeruginosa* isolates [53]. Another study on *P. acidilactici* HW01 bacteriocin showed a decrease in the production of virulence factors such as QS-regulated pyocyanin, protease, and rhamnolipid [10].

Due to the high homology of genes between nematodes and mammals and because nematodes have a relatively complete digestive system, *C. elegans* has been widely used as a host–pathogen interaction model in scientific research. In our study, the slow killing assay on *C. elegans* confirmed the anti-virulence effect of *P. pentosaceus* MZF16 against *P. aeruginosa* H103. Indeed, the presence of MZF16 has significantly enhanced the survival of *C. elegans* infected with *P. aeruginosa* H103. This result is consistent with studies showing that probiotics like *P. acidilatici* DM9 and *P. pentosaceus* SDL1409 remarkably extended *C. elegans* lifespan during *P. aeruginosa* PA14 infection [9].

## 5. Conclusions

This study highlights the potential of *Pediococcus pentosaceus* MZF16 as an effective probiotic against *Pseudomonas aeruginosa* H103. MZF16 is able to mitigate in vivo and in vitro cytotoxicity, decreases inflammation levels and pathogen adhesion and invasion in intestinal models, preserves epithelial cell integrity, and modulates *quorum-sensing* pathways, reducing virulence factor production. These findings underscore the potential of using probiotics as adjunct therapies to combat multi-drug-resistant pathogens. Further research is needed to decipher molecular mechanisms involved in these bacterial interactions.

## Figures and Tables

**Table 1:** Bacterial strains used in this study.

Strains	Genotype	References
*Pseudomonas aeruginosa* PAO1 (H103)	Wild Type	[25]
*Pediococcus pentosaceus* MZF16	Wild Type	[26]
*P. aeruginosa* H103-gfp	pHC60 (gfp, tet^R^)	This study
*P. pentosaceus* MZF16-mCherry	pRCR12 (mCherry, Cm^R^)	This study

## Data Availability

Data are contained within the article.

## Supplementary Materials

The following supporting information can be downloaded at: https://www.mdpi.com/article/10.3390/pathogens14030244/s1, Table S1: Coexistence of *P. aeruginosa* in coculture with *P. pentosaceus* MZF16.
