# Supplementary material for: Pediococcus pentosaceus MZF16 Probiotic Strain Prevents In Vitro Cytotoxic Effects of Pseudomonas aeruginosa H103 and Prolongs the Lifespan of Caenorhabditis elegans
Source: Pathogens. 2025 Mar 3;14(3):244. doi: 10.3390/pathogens14030244 (PMC11945076; doi:10.3390/pathogens14030244)
Supplement: Supplementary file 1 [file pathogens-14-00244-s001.zip › pathogens-3440827-supplementary.pdf]

Supplementary Materials

Table S1. Coexistence of *P. aeruginosa* in coculture with *P. pentosaceus* MZF16.

	***P. aeruginosa* H103 growth**
**T0**	1.3. 10^6 CFU/mL
**T24h**	8.0. 10^6 CFU/mL
